# Supplementary material for: Regnase-1-mediated regulation of neutrophils modulates SARS-CoV-2 pneumonia
Source: PLoS Pathog. 2026 Feb 9;22(2):e1013969. doi: 10.1371/journal.ppat.1013969 (PMC12912687; doi:10.1371/journal.ppat.1013969)
Supplement: Attachment — Submitted filename: Yasuda et al. Response ro Reviewers.pdf [file ppat.1013969.s014.pdf]

We would like to thank the editor and reviewers for their constructive suggestions for improving our manuscript. We have revised the manuscript and included additional new data. We have marked the parts we revised in response to the reviewers' concerns in the text.

Reviewers' Comments:

Part I - Summary

Reviewer #1: The manuscript 'Regnase-1-mediated regulation of neutrophils modulates SARS-CoV-2 pneumonia' focuses on the role of Regnase1 in SARS-CoV-2 associated lung inflammation, with a brief study on role of neutrophils conferring protection against SARS-CoV-2 infection in mice. The manuscript is easy to follow; however, the authors fail to address the link between neutrophil-mediated lung damage and protection against viral infection, which is already a big question in the field.The manuscript

Reviewer #2: This paper demonstrates that an RNase expressed in neutrophils, Regnase-1 can confer resistance to infection by a mouse-adapted SARS-CoV-2 MA-10 strain. The experimental results show that Regnase-1 haploinsufficient (Reg1$^{+/-}$) mice show reduced pneumonia severity after infection with the MA10 strain as compared to wild-type mice. Also, neutrophils were found to be elevated in Reg1$^{+/-}$ mice as compared to WT during MA-10 infection. Upon depleting the neutrophils both in Reg1$^{+/-}$ and WT mice, severe lung inflammation was observed. From this, the authors implied that Reg1 expression in neutrophils had a crucial role in regulating the resistance to MA-10 infection. Further, scRNA-seq analysis revealed four different neutrophil-subsets during the course of MA-10 infection. One of these neutrophil-subsets characterized by strong type I interferon (IFN) signatures was decreased in Reg1$^{+/-}$ mice, although there are other immune cells do show a significant role that the authors do not discuss.

With RNA-seq analysis of lung neutrophils the authors found that Reg-1 haploinsufficiency led to the upregulation of some genes associated with IFN-response signatures. One of them, Tsc22d3 exhibited significant upregulation in Reg-1 haploinsufficient neutrophils. Overexpression of Reg1 was found to suppress Tsc22d3 expression whereas a nuclease-inactive Regnase-1 failed to inhibit Tsc22d3 activity. Therefore, the findings in this paper suggest that Regnase-1 regulates neutrophil genes associated with ISG signatures by degrading certain mRNAs such as Tsc22d3. The authors also proposed that the targeted suppression of Reg1 could provide a novel

therapeutic approach for COVID-19 and other viral infections. Although, the study provides some interesting observations, the authors' analysis of the data and conclusions are not convincing. Please see my comments below.

Reviewer #3: The manuscript by Yasuda et al. uncovers how Regnase-1, involved in suppressing negative regulation of the IFN response genes, regulates neutrophils function during SARS-CoV-2 pneumonia. The authors compare the phenotype using BALB/c WT and Reg1 +/- mice and mouse-adapted SARS-CoV-2 (MA10) to investigate the role of Reg1. Reg1 +/- mice showed increased resistance to MA10 infection, exhibiting reduced body weight and inflammation with elevated neutrophil counts without any direct effect on virus gene copy number, which led them to hypothesize that neutrophils confer protection rather than directly clearing out virus infection. They validated the hypothesis by depleting the neutrophils using an anti-LY6 antibody, which resulted in increased infection and inflammation. Surprisingly, the transcriptome analysis revealed no significant differences in T cell, B cell, and NK cells between WT and Reg1 +/-. That is in coordination with comparable viral RNA levels and proinflammatory cytokines, suggesting that proinflammatory cytokine production is not the driving factor of the virus clearance. scRNAseq data revealed that cluster 3 and 4 increased at 5 dpi in both WT and Reg1 +/-; however, cluster 5 is diminished at both 3 and 5 dpi in the Reg1 haploinsufficiency model. The presence of mature neutrophil markers and absence of immature ones in both WT and Reg1 +/- suggested functional heterogeneity among the mature neutrophils rather than stages of maturation. The upregulation of proinflammatory cytokines, coupled with reduced ISGs and type I IFN-associated genes in the Reg1 +/- mice, suggests that Reg1 haploinsufficiency attenuates type I IFN response, which contributes to reduced disease severity observed in the Reg1 +/- mice. Finally, they attribute this phenotype to the upregulation of Tsc22d3, a negative regulator of Type I IFN, in the Reg1 +/- mice. The Reg1 enzymatically suppresses the Tsc22d3, particularly using the D141 residue, as D141N loses the ability to suppress the Tsc22d3.

We thank all the reviewers for carefully reading and summarizing our manuscript, for providing valuable and constructive suggestions.

Part II – Major Issues: Key Experiments Required for Acceptance

Reviewer #1: 1. Neutrophils infiltration and NETs are hallmark of lung injury in SARS-CoV-2 (and respiratory virus) infection. The authors show that depletion of neutrophils

reduces lung damage (DAD scores) while Regnase1 haploinsufficiency leads to more damage due to neutrophils infiltration. They also claim that neutrophils are protective against SARS-CoV-2 infection, with only body-weight changes, without showing replicating virus (PFU) in lungs. There is a missing link between the two phenotypes. The virus itself can cause lung damage in the absence of neutrophils, which is addressed nowhere in the study

We thank the reviewer for the important comments. In contrast to the reviewer's interpretation, our data show that neutrophil depletion increased, rather than decreased, DAD scores. To further address the reviewer's concern regarding viral burden, we examined viral RNA levels the lungs of mice infected with SARS-CoV-2 MA10 following neutrophil depletion. Noteworthy, we did not observe significant differences in the viral RNA expression among untreated WT mice, Ly6G antibody-treated WT mice, and Ly6G antibody-treated *Reg1*$^{+/-}$ mice (new Figure 2F). These results suggest that neutrophils exert a protective effect against SARS-CoV-2 MA10-induced lung damage without directly affecting viral replication in this model.

In addition, although *Reg1*$^{+/-}$ mice showed increased infiltration of neutrophils in the lungs, they showed reduced lung damage during SARS-CoV-2 MA10 infection. Neutrophil depletion in *Reg1*$^{+/-}$ mice resulted in exacerbated lung histopathology compared with neutrophil-depleted WT mice, again without altering viral RNA levels. Thus, neutrophils may play an even more pronounced protective role in *Reg1*$^{+/-}$ mice, potentially due to increased neutrophil numbers and/or qualitative changes in their functional states.

This interpretation is further supported by our single-cell RNA-sequencing analysis, which revealed neutrophil heterogeneity and characteristic transcriptional changes results are consistent with the neutrophil heterogeneity in *Reg1*$^{+/-}$ mice. Together, our data support a model in which neutrophils limit SARS-CoV-2–induced lung injury independently of viral replication, and that Regnase-1 haploinsufficiency enhances the protective capacity of neutrophils against pulmonary damage.

2. It is unclear why the authors decided to choose mouse-adapted SARS-CoV-2 strain, while the naturally occurring strains can also infect laboratory mice. It will add relevance if they use at least 1-2 strains (B.1.351 and/or emerging omicron strains) to replicate their findings.

We thank the reviewer for this concern. As a reviewer points out, several naturally occurring SARS-CoV-2 variants, including B.1.351 and omicron strains, have been reported to infect laboratory mice (Pan T. et al. Signal Transduct Target Ther 2021, Ren W. et al. mBio 2023, Ogger PP. et al. Front Immunol 2024, Kant R. et al. Viruses 2021). However, a critical distinction among these models is disease severity. While infection with the mouse-adapted MA10 strain induces severe and often lethal pneumonia in mice (Leist SR. et al. Cell 2020), Omicron variants consistently cause mild disease with limited lung pathology, as reported in the studies cited above.

Given that our study focuses on mechanisms regulating life-threatening lung injury, we believe that the MA10 strain is the most appropriate model for assessing host responses that mimic severe COVID-19. Notably, because *Reg1*$^{+/-}$ mice exhibited ameliorated pneumonia following SARS-CoV-2 infection, the MA10 model allowed us to sensitively evaluate the protective role of Regnase-1 under conditions of robust pulmonary inflammation and tissue damage.

Nevertheless, we agree with the reviewer that additional SARS-CoV-2 strains provide complementary experimental systems for investigating viral pathogenesis and host immunity. Accordingly, we now discuss the potential utility and limitations of alternative strains, including naturally mouse-tropic variants, in the Discussion section (Lines 393-401).

3. The authors should explain why they use a Regnase1 haploinsufficient (+/-) model and not a completely deficient (-/-) mouse model. Why C57Bl6 background was not used and instead BALB/c was preferred? Genotyping data is missing. Does the phenotype enhance in Regnase1-/- mice?

Reg1$^{-/-}$ (homozygote) mice spontaneously develop severe autoimmune inflammatory diseases, and most of *Reg1*$^{-/-}$ mice die by 15 weeks of age (Matsushita K. et al. Nature 2009). Therefore, we used *Reg1*$^{+/-}$ mice for infection experiments, in which the expression levels of Regnase-1 were reduced compared with wild-type mice (Tse KM et al. Sci Transl Med. 2022). Noteworthy, *Reg1*$^{+/-}$ mice do not cause inflammatory symptoms under steady-state conditions in both BALB/c and C57BL/6 genetic backgrounds.

It was demonstrated that SARS-CoV-2 MA10 infection develops severe pneumonia in BALB/c, but not C57BL/6, mice (Leist SR. et al. Cell 2020). Therefore, the use of *Reg1*$^{+/-}$ mice under BALB/c background is suitable for elucidating the role of Regnase-1 during SARS-CoV-2 infection.

4. Several quality controls are missing. E.g. Fig 1A/B/C; Fig 2D/E The authors should include mock-infected WT and Reg1+/- controls. This should partially answer comment 1 above, by comparing infected versus uninfected within WT mice.

We thank the reviewer for this comment. First, we did not observe significant differences in body weight between WT and *Reg1*$^{+/-}$ mice at the beginning of infection experiments (average body weight from day -2 to 0) shown in Figs. 1A, 1C and 2D (Figure R1 below). These results further confirm that heterozygosity of Reg1 does not affect baseline growth of mice or body weight under steady state conditions. Therefore, the body weight changes observed in Figs. 1A, 1C and 2D can be attributed to SARS-CoV-2 infection rather than to intrinsic differences between WT and *Reg1*$^{+/-}$ mice.

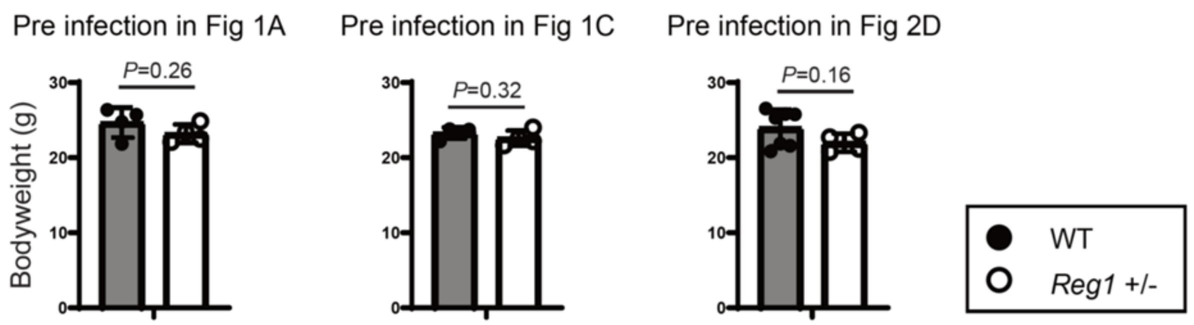

Figure R1. Body weights of WT and *Reg1*$^{+/-}$ mice at the beginning (average body weight from day -2 to 0) of infection experiments shown in Figs. 1A, 1C and 2D.

In addition, we did not detect any significant differences in the numbers of lung immune cells between uninfected WT and *Reg1*$^{+/-}$ mice (Supplementary Figure 1A).

We further examined whether PBS (Mock) treatment affected body weight. As shown in Figure R2 below, PBS treatment did not induce any body weight changes in either WT or *Reg1*$^{+/-}$ mice. Moreover, histological analysis revealed no detectable pathological changes in the lungs of WT and *Reg1*$^{+/-}$ mice 28 days after PBS treatment (new Supplementary Figure 1B).

Taken together, these results demonstrate that Regnase-1 haploinsufficiency does not cause detectable immune or pathological alterations under uninfected steady-state conditions, and that phenotypic changes observed in this study are attributable to SARS-CoV-2 MA10 infection.

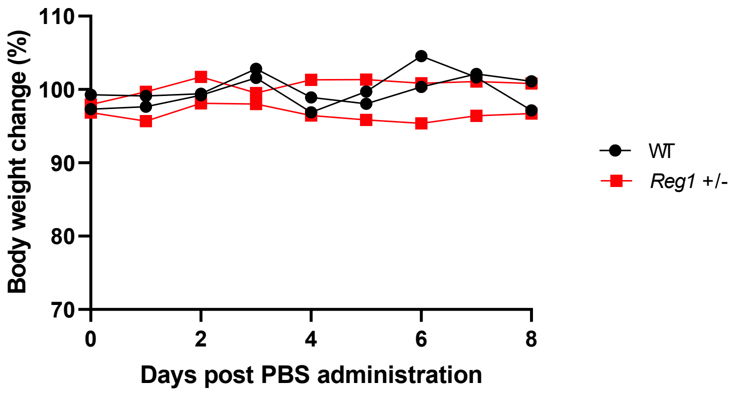

Figure R2. Body weight changes of WT and *Reg1*$^{+/-}$ mice following PBS treatment. Each line represents an individual mouse.

5. The authors should also test the expression levels of Regnase1 in different cell types upon viral infection.

We thank the reviewer for this important comment. According to the reviewer's suggestion, we analyzed the expression of Regnase-1 mRNA across multiple immune cell types in SARS-CoV-2 MA10-infected lungs by re-analyzing single cell-RNA sequence data. As shown in new Supplementary Figure S5A, the expression of Regnase-1 in neutrophils was higher than other myeloid populations including macrophages/monocytes and alveolar macrophages, and was comparable to that observed in lymphocytes. These findings are consistent with a neutrophil-intrinsic role of Regnase-1 during SARS-CoV-2 MA10 infection. Because our analysis focused on the early phase of infection (days 1-5 post infection), we expect the contribution of acquired immune cells may be limited at these time points. Nevertheless, we acknowledge that Regnase-1 expressed in acquired immune cells may contribute to disease pathogenesis during late stages of infection.

6. The authors could have used a control like Remdesivir in their assays.

We thank the reviewer for this helpful suggestion. Although a comparison between the effects of remdesivir treatment and *Reg1* haploinsufficiency in SARS-CoV-2 infection could be of interest, we feel that such an analysis is beyond the scope of this present study. Moreover, it would be difficult to make a direct and fair comparison between the effects of genetic alteration and those of pharmacological antiviral treatment.

7. Regnase1 can also degrade viral RNA, the impact of which was neither shown nor discussed, including in airway epithelial cells.

As the reviewer points out, previous studies have reported that Regnase-1 can degrade viral RNA in cell systems overexpressing Regnase-1 (Liu S. et al. Proc Natl Acad Sci USA 2013, Lin RJ. et al. J Immunol 2014). However, these studies did not demonstrate a contribution of Regnase-1-mediated viral RNA degradation to antiviral defense *in vivo*.

If Regnase-1-mediated viral RNA degradation plays a significant role during SARS-CoV-2 infection *in vivo*, one would expect *Reg1*$^{+/-}$ mice to exhibit increased viral loads and exacerbated disease. In contrast, our study showed that *Reg1*$^{+/-}$ mice developed ameliorated SARS-CoV-2 pneumonia, and we did not detect significant differences in viral loads in the lungs. These findings indicate that Regnase-1 contributes to the exacerbation of SARS-CoV-2 pneumonia *in vivo*, even though we cannot exclude the possibility that Regnase-1 may degrade SARS-CoV-2 RNA in certain cell types.

We have now discussed the potential contribution of Regnase-1-mediated viral RNA decay in the Discussion section (Lines 453-462).

8. The LD50 data of MA strain is missing which could be added in the supplementary figure.

The MLD_{50} of MA10 strain was determined by a prior experiment using wild-type Balb/c mice. However, because the vender of Balb/c mice used in our current experiments differed from that used in the previous MLD_{50} determination, we were concerned that directly applying this value might not be sufficiently accurate, even though the same mouse strain (Balb/c) was used. Therefore, to avoid potential misinterpretation, we revised the manuscript to describe viral doses uniformly in terms of $TCID_{50}$ rather than MLD_{50} throughout the manuscript. The dose of 10^2 $TCID_{50}$ used in this study corresponds to 0.3 MLD_{50} in our original description. Importantly, this change in dose notation does not affect the experimental design or the conclusions drawn from our infection experiments.

9. The authors reported increased alveolar macrophages upon infection in Regnase1+/-

mice. Though the study focuses on neutrophils, the authors should at least briefly discuss the role of alveolar macrophages and their Regnase1-dependent functions.

As the reviewer points out, *Reg1*$^{+/-}$ mice exhibited a higher number of alveolar macrophages compared with WT mice at 3 dpi. According to the reviewer's suggestion, we have added a discussion addressing the potential roles of Regnase-1 in other cell types, including alveolar macrophages, during SARS-CoV-2 MA10 infection (Lines 446-452).

10. To claim mice recovery post-infection, the assay should be extended beyond 5dpi in Fig 2D, similar to Fig 1A.

We thank the reviewer for this valuable suggestion. As pointed out, extending the observation period beyond 5 dpi would be necessary to definitively claim post-infection recovery. Because body-weight measurements in Fig. 2D were limited to 5 dpi, we have revised the Results section to avoid claiming recovery. The text has been modified to "Whereas both neutrophil-depleted WT and *Reg1*$^{+/-}$ mice exhibited continued weight loss by 5 dpi, there was a significant difference in body weight compared with untreated WT mice at 5 dpi". (Lines 202-204)

We believe this revision more accurately reflects the scope of the data presented.

Reviewer #2: Major Comments:
The manuscript aims to describe the mechanisms of SARS-CoV-2 pathogenesis that contribute to disease severity (which is known in terms of neutrophil contribution to the disease severity). The authors tried to justify the mechanistic details with decent observations, in particular, in the regulatory role of Regnase-1 during MA-10 infection in mice. However, half of these observations lack proper statistical tests and indicate the authors conclusions biased. Additionally, the paper lacks appropriate references for the previous findings and methodologies mentioned. Some of the points have been listed below:
In Figure S1A, there is an evident difference in the number of B-cells, 〖CD4〗 ^+ T-cells and neutrophils between Reg1$^{+/-}$ *and WT mice which has been overlooked by the authors. It has been stated that there is no difference in the immune cell proportions amongst the Reg1*$^{+/-}$ *and WT mice without including any statistics for the same.*

We thank the reviewers for this important and insightful comment. In the original manuscript, we reported differences in immune cell population only when they reached statistical significance. In response to the reviewer's suggestion, we have now included *p* values in all the Figures in the revied manuscript where statistical analysis was applicable.

Notably, statistical analysis revealed no significant differences in the numbers of B cells, $CD4^+$ T cells, or neutrophils between WT and *Regl*$^{+/-}$ mouse lungs shown in Figure S1.

In Figure 1E and Figure S2A, appropriate statistics should be included in order to comment about the observations made by the authors in the paper.

We thank the reviewer for this comment. As stated above, we have now included the appropriate statistical analysis and *p* values for Figure 1E and Figure S2A.

Similarly in Figure S3, the immune cell populations such as dendritic cells, monocytes, NK cells and 〖Ly6C〗^+ 〖CCR2〗^-monocytes differ in numbers between Reg1$^{+/-}$ *and WT mice but, the authors do not comment about this in the paper. There might be phenotypic changes due to the variation in these proportions which has not been noted by the authors.*

We thank the reviewers for this constructive suggestion. We have now included statistical analysis for Figure S4 (corresponding to Figure S3 in the previous version of the manuscript). As shown in the revised Figure, the numbers of dendritic cells, monocytes, or $Ly6C^+CCR2^-$ monocytes did differ statistically between *Regl*$^{+/-}$ and WT mice. In contrast, NK cells were significantly increased in *Regl*$^{+/-}$ mice compared to WT controls.

The discrepancy between the scRNA-seq and flow cytometry results for NK cells is due to differences in the timing of sample collection at 5 dpi and 6 dpi. Further studies will be required to elucidate the role of Regnase-1 in NK cells during SARS-CoV-2 MA10 infection. These points have now been described in the Results section (Lines 252-257).

The heatmap shown in Figure 4D, Ifitm3 shows an upregulation in Reg1$^{+/-}$ *mice as compared to the WT one (in the 5dpi panel) which is overlooked by the authors and,*

instead it has been generalised and mentioned that all the ISGs are downregulated (Line 264).

We thank the reviewer for this comment. As mentioned by the reviewer, *Ifitm3* expression at 5 dpi shows a modest increase in *Reg1*$^{+/-}$ mice compared with WT mice, whereas its expression is reduced at 3 dpi. According to the reviewer's suggestion, we have revised the manuscript to describe the results in a more detailed manner rather than generalizing across all ISGs.

Nevertheless, we would like to emphasize that the expression of many ISGs shows a tendency toward reduction in *Reg1*$^{+/-}$ mice overall. This trend is also supported by the GSEA of bulk RNA sequencing data shown in Fig. 4E.

In Reg-1 haploinsufficient neutrophils, apart from Tsc22d3, Fgfr1 also shows similar levels of upregulation in its expression as compared to WT mice (Figure S6A). The statistics need to be included here, and possible downregulation of the other three genes shown in the figure should be commented upon by the authors.

According to the reviewer's suggestion, we added the appropriate statistical analysis to the revised Figure S8A (correspond to Figure S6A in the original version). As shown in the revised Figure, statistical testing revealed no significant difference in the expression levels of *Fgfr1*, *Tsc22d3*, *Nr2f2*, *Axl*, and *Muc4* between WT and *Reg1*$^{+/-}$ neutrophils.

Part III – Minor Issues: Editorial and Data Presentation Modifications

Reviewer #1: 1. Line 87: 'contribute' should be changed to 'associated to'

We thank the reviewer for this suggestion. Following the reviewer's recommendation, we have changed the replaced to "associated to" in line 105 of the revised manuscript.

2. Color codes missing in Fig 4B and D.

Please find the color codes shown in Figures 4B and 4D.

3. This is not the first report on roles of neutrophils in SARS-CoV-2 and other viral infections. The authors should change the sentence in line 364.

We thank the reviewer for this comment. We have revised the sentence accordingly to "We demonstrated that neutrophils are also critical for host defense against MA10 infection." in lines 405-406 of the revised manuscript.

4. Some of the data points in Fig 5B (Reg1+/-) are shaded.

We apologize for the formatting issue in the Figure. We have corrected the presentation of the shaded data points in Figure 5B (*Reg1*$^{+/-}$).

Reviewer #2: Minor Comments:
In the discussion of the paper (line 375), appropriate references need to be included for the previous findings been mentioned.

We thank the reviewer for pointing out our mistake. We have now added appropriate references in the discussion section (Line 417).

The anti-Lys6G method used for neutrophil depletion experiments shown in the paper should be described in more detail in the methodology section. The appropriate reference of the paper first reporting this protocol has not been cited by the authors.

In accordance with the reviewer's suggestion, we have expanded the description of the anti-Ly6G–mediated neutrophil depletion method in the Methods section and added a citation to the original report describing this approach (Daley JM. et al. J Leukoc Biol 2008) (Ref. 65).

Reviewer #3: 1. The authors rely mainly on viral RNA quantification (N1, N2, ORF1b) to infer comparable viral loads. Infectious titers (e.g., plaque assay or $TCID_{50}$) from lungs would provide stronger support for the claim that Reg1 haploinsufficiency protects independently of viral clearance.

As pointed out by the reviewer, we quantified viral RNA levels rather than measuring infectious titer. We agree that assessment of infectious titer would provide stronger support for comparison of viral clearance between WT and *Reg1*$^{+/-}$ mice. However, due to limited availability of tissue samples, viral titration could not be performed. Notably, previous studies have reported a strong correlation between viral RNA levels and infectious titers (Usai C. et al. Front Microbiol. 2022), and therefore we believe that

these methodological differences do not affect the interpretation of our results.

2. Anti-Ly6G (clone 1A8) is widely used, but transient depletion can also affect some monocyte subsets. The authors should confirm depletion efficiency and specificity by flow cytometry and discuss possible off-target effects.

We thank the reviewer for this comment. The Ly6G antibody (clone 1A8) has been reported to be highly specific for neutrophils and to avoid non-specific depletion of other immune cell types (Daley JM. et al. J Leukoc Biol 2008). This specificity contrasts with that of the previously used Gr1 antibody, which recognizes both Ly6G-positive and Ly6C-expressing cells, and therefore depletes additional populations, such as monocytes (Fleming TJ. J. Immunol 1993).

Nevertheless, we further evaluated the efficacy and specificity of Ly6G antibody-mediated neutrophil depletion during viral infection. As shown in Fig. 2C, treatment with the Ly6G antibody efficiently depleted neutrophils in the lung during MA10 infection at 5 dpi. In contrast, the percentages of Ly6C^{high}CCR2^{+} monocytes among lung CD45^{+} immune cells were not decreased upon treatment with the Ly6G antibody both in WT and *Regl*$^{+/-}$ mice (new Supplementary Figure 3A). In contrast, the percentages of monocytes increased due to the depletion of neutrophils. These results demonstrate that the Ly6G antibody is highly effective and selective to deplete neutrophils.

3. Fig 2E: The DAD score which is claimed as higher upon Ly6 treatment compared to untreated, is not statistically significant. Also, the animal numbers are not same across the groups (n=3 for the untreated WT and n=4 for the treated group)

As the reviewer pointed out, the DAD score tended to be higher following Ly6G antibody treatment, although the difference was not statistically significant. Therefore, we modified the main text to describe the results more precisely.
The difference in the numbers of mice per group is because of the limited availability of age-matched mice. Although the number of mice per group varies, we believe this does not affect the interpretation of the results.

4. Figure 1C and 2B-D: While both animal experiment conditions appear similar (3MLD50), WT untreated mice displayed 100% mortality at 5 dpi in Fig 1C; however, in Fig 2D animals began to gain weight at 5 dpi.

We thank the reviewer for the comment. As shown in Figures 1C and 2D, infection experiments were conducted with the same viral load. Although we do not have a clear reason for the differences in *in vivo* infection experiments, one possible reason for the difference in severity after infection is the age of the mice. It has been shown that the SARS-CoV-2 MA10 exhibits an age-dependent increase in disease severity (Leist SR. et al. Cell 2020). Although we used 12-week-old mice in both experiments, the mice in Figure 1C were closer to 13 weeks old, and the mice in Figure 2D were closer to 11 weeks old. For each experiment, WT and *Reg1*$^{+/-}$ mice were from the same birth date. We believe this does not affect the interpretation of the results.

5. scRNA-seq pools three mice per condition/time point, treating each as one biological replicate. While practical, this limits statistical robustness. The authors should acknowledge this limitation and ideally include validation by qPCR or flow cytometry for key subset markers

We thank the reviewer for the comment. As the reviewer pointed out, in our scRNA-seq analysis, three mice were used per time point and pooled to generate a biological replicate. Owing to this experimental design, statistical comparisons between groups could not be performed, and formal statistical analyses were therefore not feasible for these datasets. We have discussed this issue as the limitation.
